# The Treatment of Eclampsia

**Published:** 1894-04

**Authors:** 


THE TREATMENT OF ECLAMPSIA.—From a new study of this important subject Dr. Charpentier, of Paris, has been led to draw the following conclusions: 1. As every albuminuric pregnant woman is liable to eclampsia, and as milk diet gives such marvelous results in albuminuria, and especially in the albuminuria of pregnancy, one should examine with the greatest care the urine of pregnant women; and if albumin be found in no matter how small a quantity, milk diet, absolute and exclusive, should be instituted at once. This is the best prophylactic treatment of eclampsia. 2. Given a case of eclampsia. If the patient is strong and vigorous and much cyanosed, begin by bleeding to the extent of 400 or 500 grammes. Then administer chloral, at the same time getting the patient to begin on milk as soon as possible. 3. If the patient is delicate, and the cyanosis less marked and the attacks less frequent, the treatment should be limited to chloral. 4. Wait for labor to come on of itself, and allow it to terminate spontaneously, whenever possible. 5. Cases of tardy labor due to uterine atony should be terminated by the forceps or version, if the child be still living; or by cephalotripsy, basiotripsy, or cranioclasis, if dead. 6. Wait before any such interference until the maternal parts are in such condition that it can be done without violence, and therefore without danger to the mother, namely, until complete dilatation of the cervix. 7. Reserve induced labor for the exceptional cases in which treatment by drugs has wholly failed. 8. Cæsarean section, accouchement force, and above all, accouchement force with deep incisions of the cervix, are to be rejected absolutely.—*Archives de Tocologie—Medical Review.*

